# Wuwei Kushen Changrong capsule alleviates DSS-induced colitis in mice via inhibition of NLRP3 inflammasome and STAT3 pathway

**DOI:** 10.3389/fphar.2024.1423012

**Published:** 2024-09-12

**Authors:** Mingjun Chen, Yang Feng, Dan Luo, Chen Zhang, Jing Zhou, Hengheng Dai, Mingxiong Lin, ZhanQi Tong

**Affiliations:** ^1^ Department of Traditional Chinese Medicine, the Second Medical Center & National Clinical Research Center of Geriatric Diseases, Chinese PLA General Hospital, Beijing, China; ^2^ Department of Oncology, the Second Medical Center & National Clinical Research Center of Geriatric Diseases, Chinese PLA General Hospital, Beijing, China; ^3^ The Third People’s hospital of longgang district, Shenzhen, China; ^4^ Tianjin Beichen Qingguang Community Healthcare Center, Tianjin, China; ^5^ Medical School of Chinese PLA, Beijing, China

**Keywords:** Wuwei Kushen Changrong capsule, murine ulcerative colitis model, NLRP3 inflammasome pathway, ulcerative colitis, STAT3

## Abstract

**Purpose:**

Wuwei Kushen Changrong capsule (Composite *Sophora* Colon-soluble Capsule, CSCC) is a Chinese patent medicine developed to treat ulcerative colitis. Studies highlight CSCC potential efficacy for ulcerative colitis (UC) but unclear mechanism limits its widely treatment for patients. We aimed to investigate the anti-colitis efficacy of CSCC and explore the mechanism by which GPR43 inhibits the NLRP3/STAT3 signaling pathway, thereby mediating the protective effects of CSCC on the intestinal barrier.

**Methods:**

The protective effects of CSCC were evaluated in a murine ulcerative colitis model induced by 3% DSS. Assessments included body weight, Disease Activity Index (DAI) score, colon length, and histopathological score. Colon tissue, cell function, and immune-inflammatory status were evaluated using immunohistochemistry, immunofluorescence, ELISA, and real-time fluorescence quantitative PCR (RT-PCR). Protein expression levels of relevant pathways and receptors were measured using Western blot. All experiments were repeated.

**Results:**

CSCC protected mice from DSS-induced colitis by upregulating Gpr43, promoting the expression of ZO-1 and Occludin tight junction proteins. Mechanistically, CSCC inhibits the MEK4/JNK1/STAT3 activation pathway, consequently suppressing the STAT3/NLRP3/IL-1β pathway and inhibiting the production of inflammatory factors such as IL-17A.

**Conclusion:**

The mechanisms through which CSCC protects against DSS-induced colitis may include upregulating Gpr43, inhibiting the STAT3/NLRP3 pathway, and suppressing inflammation factors like IL-17A. These findings highlight the mechanisms underlying CSCC’s anti-colitis effects and suggest its potential as a therapeutic candidate for managing the progression of UC.

## Introduction

Ulcerative colitis (UC) is a common inflammatory bowel disease characterized by recurrent episodes and a high risk of adverse drug reactions (Sandborn et al., 2021). Epidemiological studies have shown that the incidence rate of UC is highest in Northern European countries, estimated at approximately 24.3 cases per 100,000 people (Nanki et al., 2020). With the continuous improvement in living standards among Chinese residents, the incidence rate of UC in China has been increasing annually, reaching 11.6 cases per 100,000 people (Du and Ha, 2020). The latest consensus on the diagnosis and treatment of UC, released by the European Crohn’s and Colitis Organization (ECCO), recommends the use of 5-aminosalicylic acid, glucocorticoids, and infliximab as the main drugs for treating UC (Hibiya et al., 2017; Bischoff et al., 2020). The primary goals of treatment are to reduce the frequency of active episodes and extend the periods of remission (Rubin et al., 2019). Therefore, patients not only require lifelong medication, but also, because of long-term medication and disease progression, many patients with advanced UC develop hormone resistance and experience acute complications, such as severe bleeding or acute intestinal perforation, necessitating surgical intervention. The prognosis of these patients is usually poor (Hibiya et al., 2017).

In recent years, the interaction between G protein-coupled receptors (GPCRs) and UC has attracted considerable attention (Hou et al., 2022). Short-chain fatty acids (SCFAs) are secreted lipid components of the gut microbiota. Research has shown that SCFAs bind to the metabolic sensor G protein-coupled receptor 43 (Gpr43) and inhibit the STAT3/RORγt pathway (Xie et al., 2022). This leads to a reduction in the secretion of interleukin (IL)-1β by macrophages induced by lipopolysaccharide (LPS) and IL-4, thereby suppressing the secretion of inflammatory factors such as IL-6 and IL-17A (Dang et al., 2022; Wu et al., 2024). SCFAs play a crucial role in promoting intestinal epithelial cell regeneration, antimicrobial peptide production, goblet cell mucin secretion, and have key immunomodulatory effects in UC (Wu et al., 2024). Additionally, previous studies have shown that SCFAs have a protective effect on the intestinal mucosal barrier. They can polarize and activate the NACHT, LRR, and PYD domains-containing protein 3 (NLRP3) inflammasome pathway in the intestinal mucosa by binding to the G protein-coupled receptor Gpr43 on the intestinal epithelium (Li et al., 2023). The NLRP3 inflammasome plays a significant role in regulating intestinal inflammatory responses, in which pro-Caspase-1 is activated by the NLRP3 inflammasome to form active Caspase-1 (Larabi et al., 2020). This leads to the processing and secretion of mature IL-1β, promoting inflammation through pro-inflammatory factors in UC.

The Composite *Sophora* Colon-soluble Capsule (Chinese Pinyin translation version: Wuwei Kushen Changrong capsule, CSCC) consists of *Sophora flavescens* Aiton (Kushen), *Strobilanthes cusia* (Nees) Kuntze (Qingdai), *Bletilla striata* (Thunb.) Rchb.f. (Baiji), *Sanguisorba officinalis* L. (Diyu), *Glycyrrhiza uralensis* Fisch. ex DC. (Gancao), and the plant name has been checked with MPNS (http://mpns.kew.org, 10 March 2024). CSCC has been extensively utilized for treating UC and has received endorsement in multiple Chinese guidelines for UC treatment (Zhang et al., 2024; Kaichun et al., 2024). Phase III clinical trial results have shown that its clinical efficacy is equivalent to that of sulfasalazine tablets and non-inferior to that of mesalazine (5-ASA) sustained-release granules (Gong et al., 2012; Tong et al., 2010). Previous studies combining network pharmacology with ultra-high performance liquid chromatography-quadruple time-of-flight mass spectrometry (UHPLC-Q-TOF/MS) technology have identified the active metabolites of CSCC and predicted that the main pathway in the treatment of UC is the IL-17 signaling pathway (Ding et al., 2020; Chen et al., 2020). CSCC employs pH-dependent oral colon-targeted drug delivery technology to preserve intestinal mucosal homeostasis. Nonetheless, the exact mechanism by which CSCC modulates intestinal mucosal immunity and sustains the integrity of the intestinal mucosal barrier in UC to attain therapeutic efficacy remains elusive. In this study, we aimed to investigate the anti-colitis efficacy of CSCC and explore whether and how Gpr43 inhibits the STAT3/NLRP3 signaling pathway, thereby mediating the protective effects of CSCC on the intestinal barrier.

## Materials and methods

### Reagents

Our research group used UHPLC-Q-TOF/MS to analyse the metabolites of CSCC, considering that matrine, ammothamnine, sophora flavescens neoalcohol J, and Sophora oxytol U as main metabolites of CSCC (Chen MJ. et al., 2023). CSCC (batch number: C1020210202) was supplied by Coway Pharmaceutical Co., Ltd. (Beijing, China). The metabolites in CSCC have been published in our previous studies (Table 1) (Tong et al., 2010; Ding et al., 2020; Chen et al., 2020). Pentobarbital sodium (batch number: P3761) was purchased from Sigma Merck. 5-aminosalicylic acid (batch number: A129982) was purchased from Aladdin. Dextran sulfate sodium salt (DSS) with a molecular weight of 36,000–50,000 Da (batch number: D8906) was purchased from Sigma. Dulbecco’s Modified Eagle Medium (DMEM; batch number SH30022.01B) was purchased from HyClone. Fetal bovine serum (FBS) was purchased from Gibco (batch number 10270-106). Phosphate-buffered saline (PBS) was purchased from SolarBio (batch number: P1010). 0.25% Trypsin (batch number T1350) was purchased from SolarBio. Formaldehyde (batch number 10010018) was purchased from China National Pharmaceutical Group Chemical Reagent Co., Ltd. Triton X-100 (batch number T8210) was purchased from SolarBio. 5% bovine serum albumin (BSA) blocking solution (batch number: A8010) was obtained from SolarBio. Protein Marker Helix (batch number P12103) was used as the protein marker. Sodium dodecyl sulfate (SDS) (batch number: S8010) was purchased from SolarBio. Tetramethylethylenediamine (TEMED) (batch number: T8090) was purchased from SolarBio. Ammonium persulfate (batch number A1030-1) was purchased from SolarBio. Polyvinylidene fluoride (PVDF) membrane (batch number IPVH00010) was purchased from Millipore. Chemiluminescent substrate (batch number WBKLS0500) was purchased from Millipore. Tween-20 (batch number T8220-100) was purchased from SolarBio. Radioimmunoprecipitation assay (RIPA) (Strong) rapid lysis buffer for tissue/cell (batch number R0010) was purchased from SolarBio. BCA Protein Concentration Assay Kit (batch number PC0020) was purchased from Solarbio. Eosin Y (batch number E8090) was purchased from SolarBio. Neutral resin (batch number: G8590) was purchased from SolarBio. Safranin (batch number G1004) was purchased from Servicebio. Anti-fade mounting medium for fluorescent staining (batch number S2110) was purchased from SolarBio. Blocking solution using goat serum (batch number SL038-10 mL) was purchased from SolarBio. 20x Tris-EDTA antigen retrieval solution (pH 9.0) (batch number G1203) was purchased from Servicebio.

**TABLE 1:** Formula of CSCC.

Latin names	English names	Chinese names	Medicinal part	Weight(g)
*Sophora flavescens* Aiton	root of Light yellow *sophora*	Kushen	Dry root	15
*Strobilanthes cusia* (Nees) Kuntze	Indigo Naturalls	Qingdai	Dry leaves	3
*Bletilla striata* (Thunb.) Rchb.f	Bletillae rhizoma	Baiji	Dry root	3
*Sanguisorba officinalis* L	Sanguisorbae Radix	Diyu	Dry root	10
*Glycyrrhiza uralensis* Fisch. ex DC.	Glycyrrhizae Radix Et Rhizoma	Gancao	Dry root	10

Rabbit polyclonal antibody against Gpr43 (batch number NBP3-12190) and RORγt (batch number NBP2-24503) was obtained from Novus. Rabbit polyclonal antibody against NLRP3 (batch number PAB37930), STAT3 (batch number PAB46077), Occludin (batch number PAB36669), Zonula occludens-1(ZO-1) (batch number PAB31141), MEK1 (batch number PAB36512), MKK4 (batch number PAB32161), JNK1 (batch number PAB30101), Goat anti-Rabbit IgG secondary antibody (batch number SAB43714) and glyceraldehyde 3-phosphate dehydrogenase (GAPDH) (batch number PAB36269) was purchased from Bioswamp. Phosphorylated transducer and activator of transcription-3 (P-STAT3) rabbit polyclonal antibody (batch number: Ab32143) was purchased from Abcam. Enzyme-linked immunosorbent assay (ELISA) kits for Mouse IL-17A (batch number MU30386), tumor necrosis factor α (TNF-α) (batch number MU30030), IL-1β (batch number MU30369) and Caspase-1 (batch number MU31304) were purchased from Bioswamp.

### Animal experiments

Male C57BL/6N mice weighing 20–22 g was used in this study, with 32 experimental mice and 2 quarantine mice. The animals were sourced from Charles River Ltd. (Beijing, China). The housing environment maintained a temperature of 22°C–26°C, relative humidity of 50%–60%, and artificial light on a 12-h light-dark cycle in the Laboratory Animal Center of the Academy of Military Medical Science (AMMS) of China. Prior to the experiments, the mice underwent an acclimatization period of 3–7 days. All experiments were conducted in accordance with the National Institutes of Health Guide (NIH) for the Care and Use of Laboratory Animals (NIH Publication No. 8023) and approved by the Ethics Committee of the Chinese PLA general hospital (No. S2022-484-01).

### Induction and therapies of colitis

**FIGURE 1:**
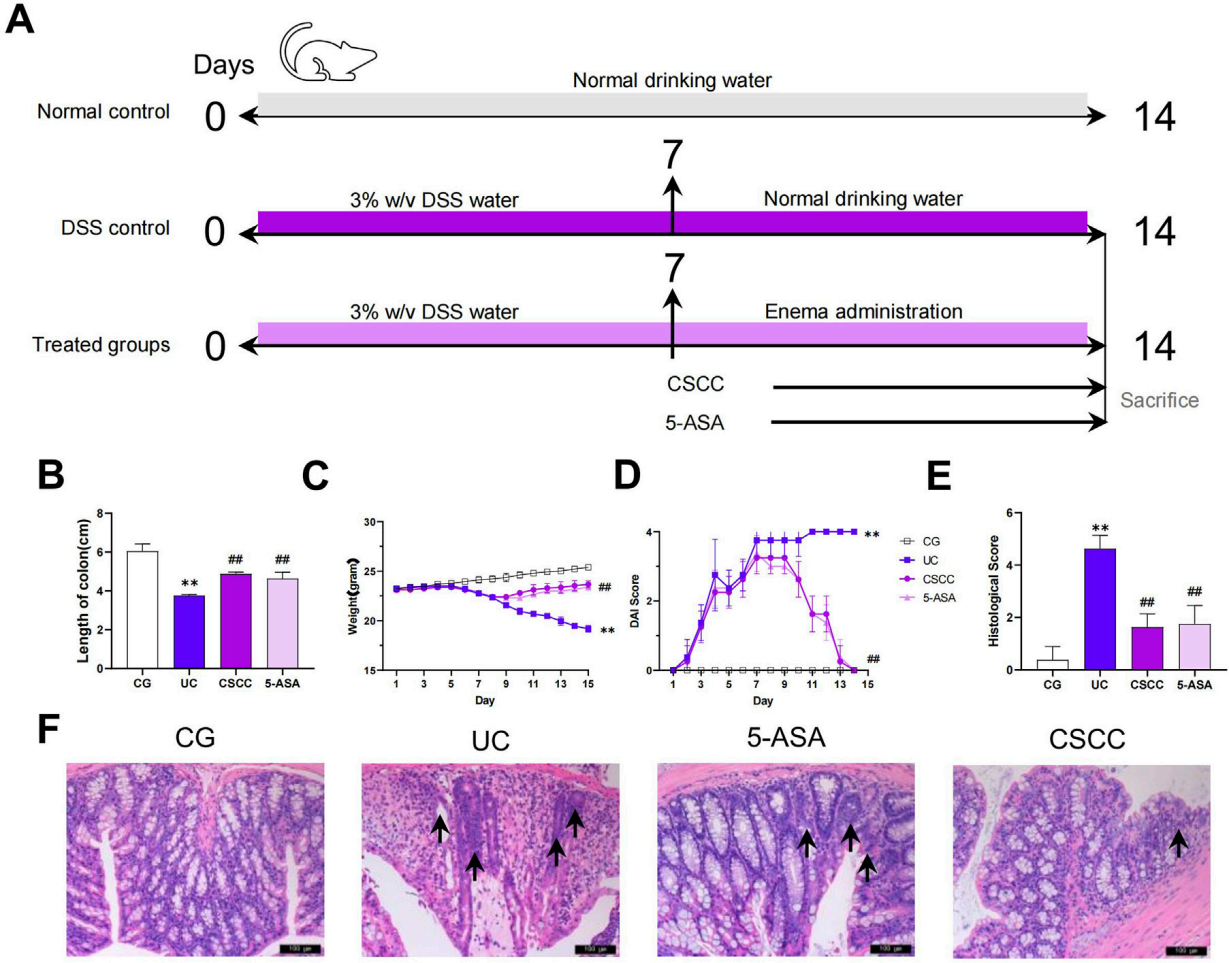
Therapeutic effects of CSCC on UC murine model. **(A)** Design of experiments of UC murine model and treatment. Relative changes in **(B)** length of colon, **(C)** body weight, **(D)** DAI score and **(E)** histological colonic score. **(F)** Relative changes in pathological observation (H&E staining, 200 ×, scale bar = 100 μm) of colon tissue sections, and arrows mark incomplete tissue, irregular glands, and numerous inflammatory cell infiltrates. Data were presented as mean ± SD (n = 6). ^#^^#^ *p* < 0.01, ^#^ *p* < 0.05 vs UC group. **p < 0.01, *p < 0.05 vs control group. ns: no statistical significance.

The mice were randomly divided into four groups, including control, UG (ulcerative colitis model group), 5-ASA, and CSCC group, with eight mice in each group. Except for the control group, which had free access to distilled water, the remaining mice were given free access to 3% DSS for 7 days to induce colitis (Figure 1A). After 7 days, the control and UG groups were given an equal amount of 0.9% saline enema, whereas the 5-ASA group received a daily enema dose of 0.5 g/kg × M of ultrafine mesalazine (5-ASA) powder (g), prepared in 0.2 mL of distilled water enema solution (M: mouse body weight, unit: kg). The CSCC group was administered a daily enema dose of 0.8256 g/kg × M of ultrafine CSCC suspension. This suspension was meticulously prepared with 0.2 mL distilled water to form the enema solution (Ding et al., 2020; Chen MJ. et al., 2023). Throughout the experiment, the mice continued to drink 3% DSS. After 7 days of intervention, all mice were euthanized using 1% pentobarbital sodium, and the colon tissues and feces were collected. If any animals were found alive, they were anesthetized with 100 mg/kg pentobarbital sodium until there was no heartbeat or respiration to confirm death.

### Disease Activity Index (DAI) assessment

Mice were monitored daily, with measurements taken every 2 days to assess changes in body weight, stool consistency, and the presence of fecal occult blood. The DAI scores, calculated based on a grade scale of 0–4 for parameters were used to evaluate the severity of colitis (Ma et al., 2023).

### Hematoxylin and eosin staining and histopathologic analysis

The distal colon tissue located 1.0 cm from the end was fixed in 4% paraformaldehyde solution, dehydrated in a graded ethanol series, cleared in xylene, embedded in paraffin, and sectioned at a thickness of 3 μm. The sections were then floated on a water bath, placed onto glass slides, carefully adhered to the slides, and stained with hematoxylin and eosin (H&E). The severity of colitis was assessed based on lesion scores determined through histological evaluation of the colon tissue, as previously described (Zhang et al., 2022).

### Drug-containing serum preparation

In the present study, drug-containing serum was prepared according to the method described previously by Qu et al. (Qu et al., 2023). Male Wistar rats (8 weeks of age, approximately 250 g) were obtained from Charles River Ltd. (Beijing, China) and randomly assigned to three distinct groups. The first group served as the model group, receiving an equivalent volume of saline via gavage. The second and third groups were administered CSCC and 5-ASA solutions, respectively, at concentrations of 432 mg/mL and 100 mg/kg, with a gavage volume of 10 mL/kg, once daily for seven consecutive days. Following the final administration, blood was aspirated from the abdominal aorta of fasted rats, which had been deprived of food (but not water) for 12 h. The collected blood was allowed to rest for 30 min before being centrifuged at 4°C at 3,000 r/min for 15 min. The resultant supernatant, designated as CSCC serum and 5-ASA serum, was then processed identically. Both the model and medicinal serums were subjected to inactivation by heating at 56°C for 30 min, filtered through a 0.22-micron sterile filter, and stored at −80°C until further use. These serums were utilized in subsequent *in vitro* experiments, with the control serum serving as the blank control, and the medicinal serums diluted to appropriate concentrations as required.

### Cell culture and treatment

The RAW264.7 cell line (obtained from the Shanghai Cell Bank, Chinese Academy of Sciences) was cultured in complete medium containing 10% FBS. After 7 days of incubation, the cells were divided into different treatment groups as follows: Cells in the control group were cultured for 24 h and then treated with a combination of physiological saline and drug-containing serum; whereas cells in the model, CSCC, and 5-ASA groups were first exposed to LPS (Sigma, CA, USA) for 24 h before receiving specific treatments for an additional 24 h.

### Immunofluorescence

Immunofluorescence detection was performed on mouse colonic tissue and cell samples in a 12-well plate fixed in 4% paraformaldehyde (PFA) for 8–12 h. The fixed tissues were rehydrated in a 30% sucrose solution for 24 h, followed by slicing colonic tissue samples in a cryostat and staining them with primary antibodies (NLRP3, Gpr43, and MEK4). After washing out the primary antibodies with TBS containing 0.025% Triton three times, secondary antibodies were applied at room temperature for 2 h in the dark. The slices were rinsed with TBS for 10 min, incubated with DAPI solution for 5 min at room temperature, washed three more times for 10 min each, and imaged using a Leica inverted fluorescence microscope (Leica, DMIL LED).

### ELISA analysis

The colon segments were rinsed with PBS to remove excess blood, and the colonic homogenate and cell protein samples were centrifuged at 10,000 × g for 5 min. Protein levels of IL-17A, TNF-α, IL-1β and Caspase-1 were analyzed using mouse-specific ELISA kits (Bioswamp, China) following the manufacturer’s instructions.

### Western blot analysis

The supernatant from the colonic tissue was used for western blotting following the same preparation method as that used for ELISA. The protein concentration was determined using the classical BCA protein assay. SDS-polyacrylamide gel electrophoresis (PAGE) gels were prepared, samples were loaded and electrophoresed, then transferred to PVDF membrane. The membrane was blocked with 5% skim milk for 1 h, incubated with primary antibodies overnight at 4°C, washed with tris-buffered saline and Polysorbate 20 (TBST), and incubated with secondary antibodies (1:2000) for 1 h at room temperature, followed by electrochemiluminescence (ECL) detection. The membrane was then analyzed using a fully automated chemiluminescence imaging system (Tanon-5200, Shanghai, China), and the grayscale values of the relevant bands were read using TANON GIS software.

### Quantitative RT-PCR

Colonic total RNA was prepared using TRIzol (Invitrogen), followed by reverse transcription into cDNA (Essence Biotech, China). Quantitative PCR (qPCR) was then performed using a LightCycler (Roche) and SYBR Green Supermix (Essence Biotech, China) with a reaction volume of 20 μL. The specific primers used were as follows: IL-1β 5′-GTG​GCA​GCT​ACC​TGT​GTC​TT-3′ and 5′-GGA​GCC​TGT​AGT​GCA​GTT​GTT-3′, NLRP3 5′-CCT​TAA​GCT​GGA​GCT​GCT​GTC-3′ and 5′-TCACCTCTCGGCA GTGGATA-3′, ACTB 5′- GTG​CTA​TGT​TGC​TCT​AGA​CTT​CG-3′ and 5′-ATG​CCA​CAG​GAT​TCC​ATA​CC-3'.

### Statistical methods

Continuous variables were reported as the mean ± standard deviation. The data analysis was performed using GraphPad Prism 9.0 software (GraphPad Software, Inc.). One-way analysis of variance (ANOVA) was used for multiple comparisons between groups, whereas the Student’s t-test was used for pairwise comparisons between two groups. *p* values less than 0.05 were considered statistically significant.

## Results

### CSCC significantly alleviated signs of DSS-induced chronic colitis in mice

CSCC effectively improved the signs and histopathology of acute colitis. Compared with the control group, mice in the DSS group exhibited significant weight loss, diarrhea, and rectal bleeding. The severity of colitis was assessed by measuring the body weight, colon length, and infiltration of inflammatory cells. Considering that colon shortening is a key indicator of DSS-induced colitis in mice, we evaluated colon length in mice with colitis treated with CSCC (Kuttke et al., 2022). The colon length was markedly decreased in mice with DSS-induced colitis compared with that in the control group ((Figure 1B, *p* < 0.01). Furthermore, compared with the DSS group, mice treated with CSCC and 5-ASA showed a significant increase in colon length (*p* < 0.01). Both 5-ASA and CSCC significantly alleviated DSS-induced weight loss (Figure 1C). The DAI, which assessed the severity of intestinal inflammation based on weight loss, stool consistency, and rectal bleeding, was significantly elevated in the DSS group, but was markedly reduced by administering 5-ASA and CSCC (Figure 1D). Consistent with these results, both the 5-ASA and CSCC groups had reduced histopathological damage, including mucosal epithelial injury, alterations in crypt number and structure, reduction in goblet cells, and inflammatory cell infiltration, leading to lower histopathological scores than those in the DSS group (Figure 1E). In summary, 5-ASA and CSCC administration ameliorated the signs and histopathology of DSS-induced colitis.

### CSCC strengthens the intestinal barrier function in mice with colitis

**FIGURE 2:**
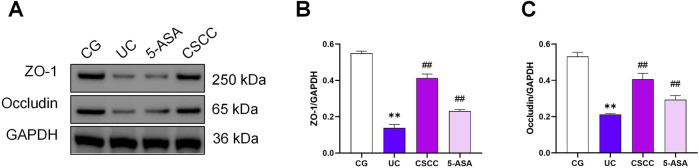
Therapeutic effects of CSCC on the intestinal mucus barrier. **(A, B)** ZO-1 and **(C)** Occludin protein redistribution in different groups were quantified by Western blot. Data were presented as mean ± SD (n = 6). ^##^ *p* < 0.01, ^#^ *p* < 0.05 vs UC group. **p < 0.01, *p < 0.05 vs control group. ns: no statistical significance.

Enhanced intestinal integrity and permeability improves the mechanical barrier function of the intestine. ZO-1 and Occludin, members of the tight junction protein family, are associated with intestinal integrity in colonic tissue (Deng et al., 2022). In our study, we performed immunohistochemistry to demonstrate the expression of ZO-1 and Occludin in colonic tissue. Figure 2 shows that ZO-1 and Occludin levels were significantly decreased in the DSS group compared with the control group, whereas the administration of 5-ASA and CSCC effectively restored the protein expression of ZO-1 and Occludin. These results suggest that CSCC and 5-ASA can restore the functionality of ZO-1 and Occludin in colitis, thereby restoring the intestinal mucosal barrier.

### CSCC suppresses DSS-induced NLRP3 inflammasome activation in colitis

**FIGURE 3:**
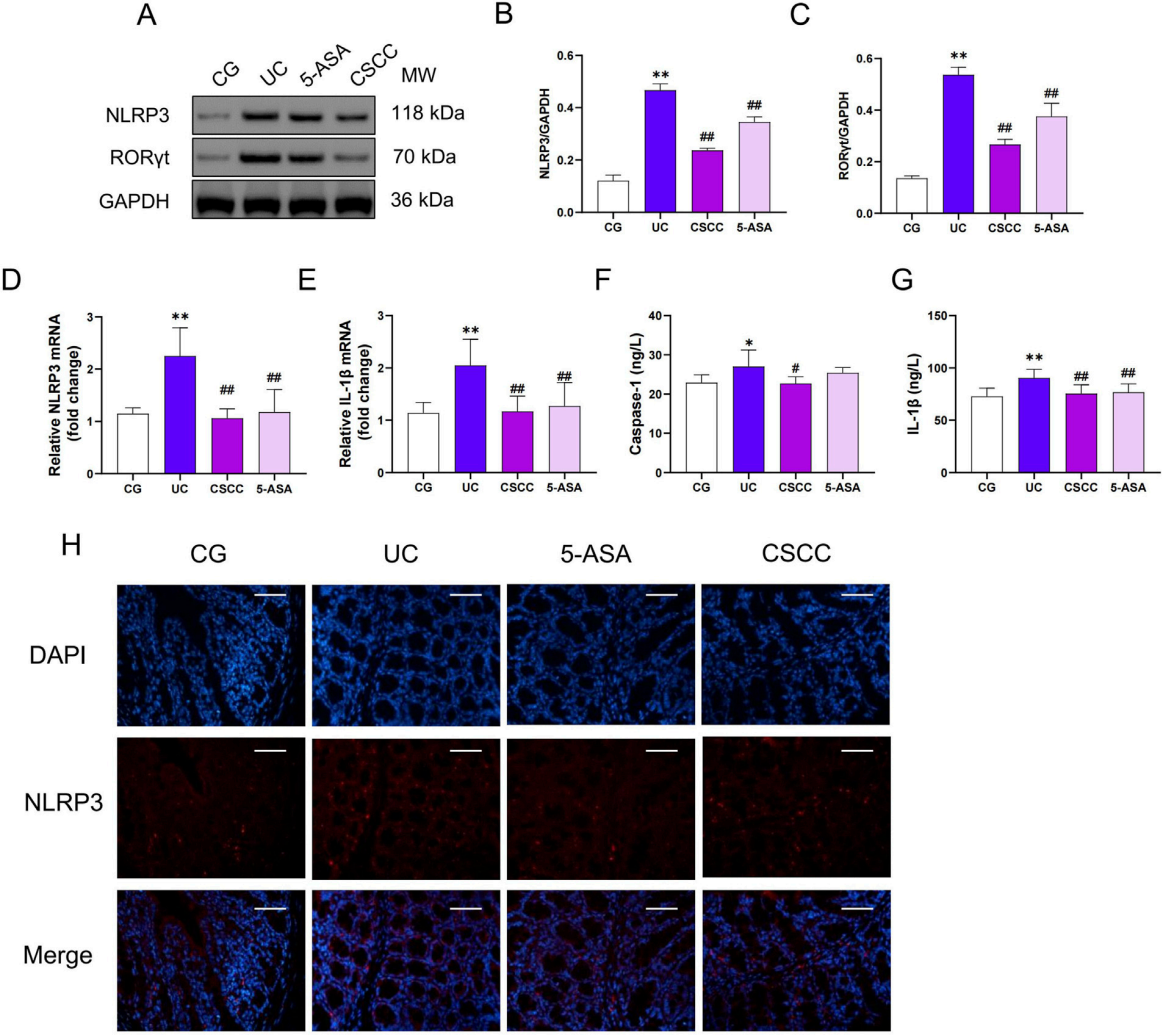
CSCC suppressed the NLRP3 Inflammasome Expression **(A)** Relative quantification of western blot in colonic tissue was performed for protein redistribution of **(B)** NLRP3 and **(C)** RORγt. RT-qPCR was performed to analyze the mRNA levels of **(D)** NLRP3 and **(E)** IL-1β. ELISA was performed to analyze the proinflammatory cytokines **(F)** IL-1β and **(G)** Caspase-1. **(H)** Levels of NLRP3 were tested by Immunofluorescence staining in colonic tissue (200 ×, scale bar = 100 μm). Data were presented as mean ± SD (n = 6). ^##^ *p* < 0.01, ^#^ *p* < 0.05 vs UC group. **p < 0.01, *p < 0.05 vs control group. ns: no statistical significance.

NLRP3 is a key inflammasome responsible for the generation of Caspase-1 and IL-1β, which are crucial for initiating intestinal inflammation (Larabi et al., 2020). Therefore, we measured the relative expression of NLRP3 and RORγt in colonic tissues. As shown in Figure 3, compared with the control group, the relative expression of NLRP3 and IL-1β was significantly increased in the DSS group, both at the mRNA and protein levels (*p* < 0.01). In contrast, CSCC intervention significantly reduced the mRNA and protein expression levels of NLRP3, RORγt, and IL-1β in colonic tissues (*p* < 0.01). Additionally, CSCC enhanced Caspase-1 expression (*p* < 0.05), whereas no significant difference in Caspas-1 expression was observed in the 5-ASA group. These results indicate that CSCC suppresses the generation of Caspase-1 and IL-1β by reducing the production of NLRP3.

### CSCC significantly reduced the activation of the STAT3 signaling pathway

**FIGURE 4:**
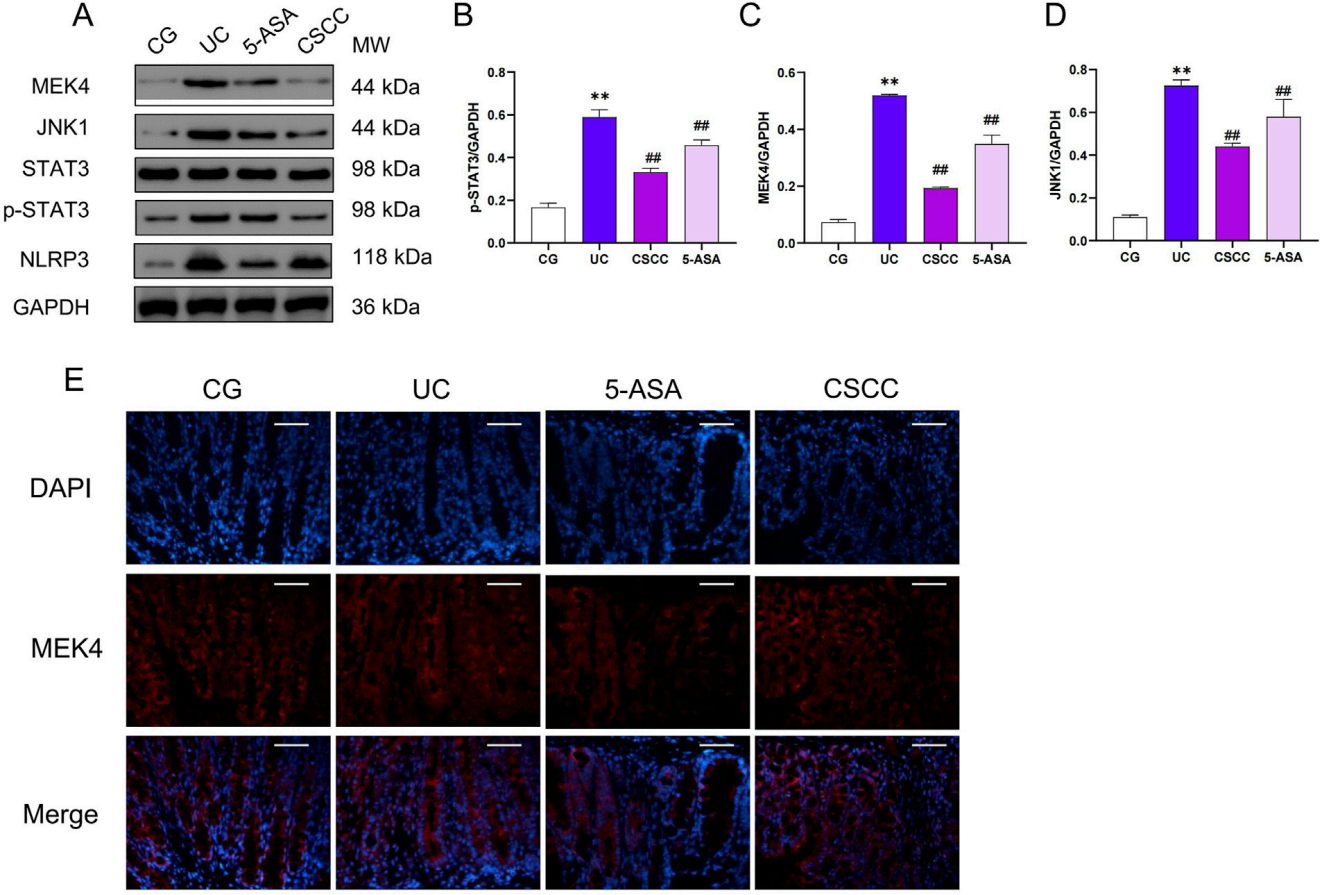
The effects of CSCC on the inhibition of the STAT3 signaling pathway. **(A)** Relative quantification of western blot in colonic tissue was performed for protein redistribution of **(B)** p-STAT3, **(C)** MEK4, and **(D)** JNK1. **(E)** Levels of MEK4 were tested by Immunofluorescence staining in colonic tissue (200 ×, scale bar = 100 μm). Data were presented as mean ± SD (n = 6). ^##^ *p* < 0.01, ^#^ *p* < 0.05 vs UC group. **p < 0.01, *p < 0.05 vs control group. ns: no statistical significance.

Given the regulatory role of the STAT3 signaling pathway in NLRP3 activation, we hypothesized that STAT3 activation might be a key pathway through which CSCC modulates NLRP3 (Lu et al., 2024). Therefore, we investigated the STAT3 signaling pathway and assessed the activity of the STAT3 pathway in mice with DSS-induced colitis. Following the intervention, there was a significant upregulation of MEK4, JNK1, and pSTAT3 protein levels in the DSS group compared with the control group, as shown in Figure 4. Additionally, through immunofluorescence and protein level analysis, we observed that CSCC and 5-ASA significantly downregulated the expression of pSTAT3 in the STAT3 signaling pathway, indicating that CSCC can regulate the STAT3 signaling pathway.

### CSCC alleviated colitis specifically by activating Gpr43

**FIGURE 5:**
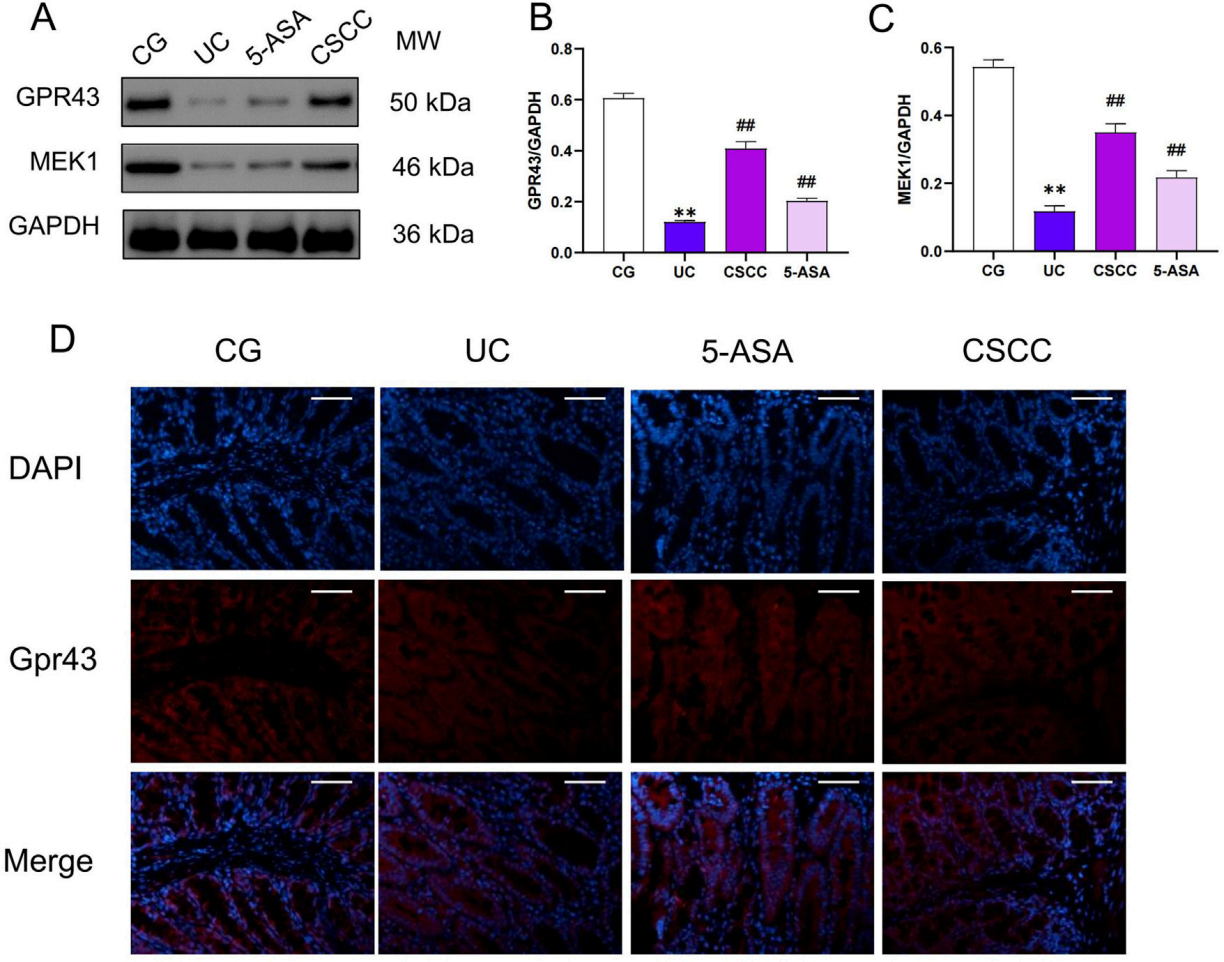
CSCC effectively activated the production of Gpr43. **(A)** Relative quantification of western blot in colonic tissue was performed for protein redistribution of **(B)** Gpr43 and **(C)** MEK1. **(D)** Levels of Gpr43 were tested by Immunofluorescence staining in colonic tissue (200 ×, scale bar = 100 μm). Data were presented as mean ± SD (n = 6). ^##^ *p* < 0.01, ^#^ *p* < 0.05 vs UC group. **p < 0.01, *p < 0.05 vs control group. ns: no statistical significance.

In our previous study, we found that CSCC significantly increased the production of the intestinal microbiota metabolite butyrate, which regulates intestinal inflammation by stimulating G-protein-coupled receptor 43 (Gpr43) and helps maintain intestinal epithelial barrier function (Li et al., 2023). In this study, immunofluorescence and western blotting analysis revealed that after DSS intervention, the expression of Gpr43 and downstream MEK1 protein were significantly reduced compared with that in the control group (Figures 5A–C). However, in the CSCC and 5-ASA groups, the protein levels of Gpr43 were significantly higher (*p* < 0.01) than those in the DSS group. Similarly, immunofluorescence results showed a significant increase in Gpr43 expression levels in the CSCC group (Figure 5D). These results indicate that by upregulating the Gpr43/MEK1 signaling pathway, CSCC plays a role in regulating intestinal barrier function to protect the colon from damage caused by autoimmune inflammation.

### Impact of CSCC on DSS-induced mouse STAT3/NLRP3 inflammasome pathway

**FIGURE 6:**
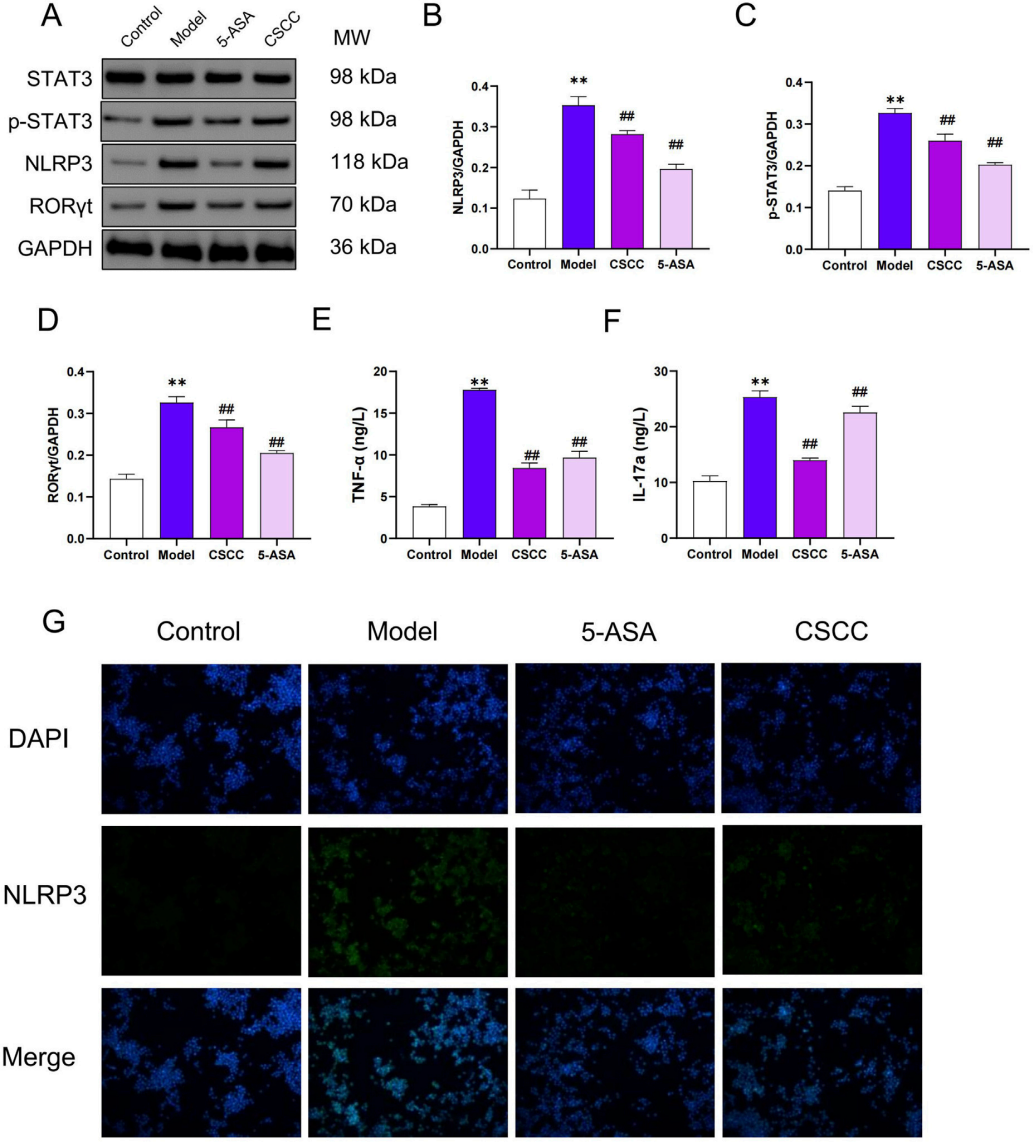
Effects of CSCC on STAT3/NLRP3 in RAW264.7 cells **(A)** Relative quantification of western blot in RAW264.7 cells was performed for protein redistribution of **(B)** NLRP3, **(C)** p-STAT3 and **(D)** RORγt. ELISA was performed to analyze the proinflammatory cytokines **(E)** TNF-α and **(F)** IL-17A. **(G)** Levels of NLRP3 were tested by Immunofluorescence staining in colonic tissue (200 ×, scale bar = 100 μm). Data were presented as mean ± SD (n = 6). ^##^ *p* < 0.01, ^#^ *p* < 0.05 vs UC group. **p < 0.01, *p < 0.05 vs control group. ns: no statistical significance.

Based on the results of the *in vitro* experiments, we examined the protein expression levels of NLRP3, and STAT3 in RAW264.7 cells. Compared with the model group, these proteins were significantly reduced after the administration of CSCC (Figures 6B,C). ELISA and Western blotting results indicated that CSCC significantly downregulated the expression levels of NLRP3 and pSTAT3 compared with the model group, and markedly inhibited the elevation of TNF-α and IL-17A (Figures 6E,F). Immunofluorescence showed that LPS treatment significantly promoted activation of the STAT3/NLRP3 signaling pathway and subsequent inflammasome activation (Figure 6G). Considered together, these data suggest that CSCC-induced inhibition in colitis is closely associated with the suppression of the STAT3/NLRP3 signaling pathway.

## Discussion

Currently, the first-line treatments for UC are drugs such as mesalazine, steroids, and immunosuppressants, which cause long-term mental and physical burden to patients (Sandborn et al., 2021). Multiple studies have demonstrated that Traditional Chinese medicine (TCM), significant potential and advantages in regulating intestinal mucosal immunity; however, the molecular mechanisms through which TCM regulates intestinal mucosal immunity are unclear and this limits their promotion for treating UC (Prayoga et al., 2024).

The proactive development of novel medications grounded in TCM principles offers a promising therapeutic avenue for UC. CSCC, which leverages pH-dependent oral colon-targeted drug delivery technology, stands as a testament to this approach. Having received approval for the treatment of ulcerative colitis and earning widespread acclaim for its effectiveness, CSCC has garnered widespread recognition for its efficacy (Zhang et al., 2024). Results from multicenter phase II and III clinical trials have shown that CSCC is non-inferior to mesalazine delayed-release granules in terms of clinical efficacy for treating UC, and it is associated with greater improvement in mucosal histology compared with the control group (Tong et al., 2010; Chen et al., 2020). As a Chinese patent medicine for the treatment of active UC (classified as damp-heat accumulation syndrome in TCM), CSCC has already been included in the reimbursement drug list (Langhorst et al., 2015). Previous studies have shown significant inhibitory effects on invasive intestinal bacteria such as *Escherichia coli* and *Bacteroides fragilis*, a significant increase in the production of SCFAs such as butyrate as intestinal microbiota metabolites, and immunosuppressive effects on intestinal epithelial IL-1β and IL-17 (Ding et al., 2020; Chen MJ. et al., 2023). While understanding of the mechanism by which CSCC regulates intestinal mucosal immunity is limited. As a quintessential TCM formula, the precise identification of its primary active metabolites and the exploration of its potential drug targets are essential areas that warrant further scrutiny.

Butyrate, the main metabolite produced during the fermentation of carbohydrates by protective intestinal bacteria, is significantly reduced in the intestines of patients with active UC, and its levels are closely related to the decreased content of its primary producing bacteria (such as *Lactobacillus* and *Bifidobacterium*) in the intestines (Liu et al., 2023). Multiple studies have shown that dysbiosis and immune dysregulation in the gut can lead to intestinal mucosal dysfunction by promoting the secretion of pro-inflammatory cytokines such as IL-1β, IL-6, and IL-17A (Liu et al., 2019; Chen X. et al., 2023). Under normal intestinal mucosal immune homeostasis, probiotics and their metabolite, butyrate, promote the production of IL-10 by Treg cells and IL-22 by NCR^+^ ILC3, thereby enhancing the production of intestinal tight junction proteins (Occludin, ZO-1), promoting intestinal mucosal cell regeneration, and improving inflammatory responses in UC (Li et al., 2022). This study demonstrates that DSS-induced colitis leads to a decrease in Occludin and ZO-1 proteins. However, both 5-ASA and CSCC upregulated the expression of intestinal tight junction proteins. These findings further support the protective role of CSCC in DSS-induced colitis.

Further research has shown that butyrate binds to the metabolic sensor Gpr43, which stimulates the synthesis of endogenous host defense peptides (HDP) in intestinal epithelial cells through mitogen-activated protein kinase 1 (MEK1)/extracellular signal-regulated kinase (ERK) pathways and cell growth pathways (Liu et al., 2023). This process plays an important regulatory role in promoting intestinal mucosal immune homeostasis and can serve as a potential target for drug intervention. Although the function of Gpr43 has been extensively studied in the intestines, immune cells, and fat cells, recent research has found that the regulation of Gpr43 expression is induced by inflammatory factors such as LPS, TNF-α, and granulocyte-macrophage colony-stimulating factor (GM-CSF) (Liu et al., 2024; Gao et al., 2023). These factors modulate the expression of Gpr43 in human monocytes through the transcription factor X-box binding protein 1 (XBP1), leading to the activation of the MEK4/JNK1 pathway and promotion of STAT3 phosphorylation (Mielczarek-Lewandowska et al., 2019). In a study on DSS-induced colitis in mice, CSCC exhibited powerful anti-inflammatory effects by inhibiting the JNK1/STAT3 signaling pathway, reducing the levels of pro-inflammatory cytokines TNF-α, IL-1β, and IL-17.

These results were confirmed using *in vitro* data. LPS-induced secretion of IL-17a and TNF-α, which inhibited Gpr43 protein expression, was significantly suppressed by CSCC. CSCC effectively suppressed the expression of the p-STAT3/NLRP3 signaling pathway and promoted the upregulation of Gpr43. Additionally, CSCC inhibited the elevated production of RORγt induced by LPS, thus suppressing inflammatory cell differentiation. Both *in vivo* and *in vitro* experimental results demonstrate that CSCC alleviates DSS-induced colitis in mice by regulating STAT3 activation through Gpr43.

STAT3 is a critical transcriptional regulator of apoptosis, cell death, and inflammation. Activation of STAT3 interacts with the NLRP3 promoter, subsequently stimulating apoptosis and cell death (Chen X. et al., 2023; Ratsimandresy et al., 2017). A previous study confirmed that pSTAT3 upregulates NLRP3 by directly enhancing the acetylation of histones H3 and H4 at the NLRP3 promoter in macrophages treated with LPS (Tan et al., 2024). NLRP3 plays a crucial role in local inflammation (Xue et al., 2022). Activated Caspase-1 acts downstream of the NLRP3 inflammasome, leading to the cleavage of pro-interleukin-1β (pro-IL-1β) to generate mature IL-1β (Xue et al., 2022). Increasing evidence suggests that limiting NLRP3 inflammasome and JNK1/STAT3 signaling contributes to the alleviation of intestinal inflammation. Inflammatory cells aggregate and secrete pro-inflammatory cytokines like interleukin-6 (IL-6) and TNF-α, further stimulating and exacerbating the JNK1/STAT3 signaling pathway, leading to intestinal inflammation (Zhang et al., 2021). According to our previous research, CSCC can inhibit the differentiation of pro-inflammatory cells such as Th17 and Lti ILC3 and suppress the secretion of IL-1β and IL-17A (Chen MJ. et al., 2023). Therefore, we hypothesize that CSCC is involved in inhibiting STAT3/NLRP3, thereby suppressing the production of pro-inflammatory cytokines Caspase-1 and IL-1β, and restoring intestinal mucosal immune homeostasis. In this study, inflammasome-associated factors, including p-STAT3, NLRP3, IL-1β, and caspase-1, were significantly enhanced in a DSS-induced mouse colitis model. However, treatment with CSCC significantly reduced their expression. Additionally, our research showed that CSCC significantly increased the expression of tight junction proteins, such as ZO-1 and Occludin, thereby enhancing intestinal immune barrier function. Furthermore, we demonstrated that Gpr43/MEK1 exacerbates the reduction of inflammasome induced by CSCC. This indicates that CSCC may regulate STAT3/NLRP3 through Gpr43.

## Conclusion

**FIGURE 7:**
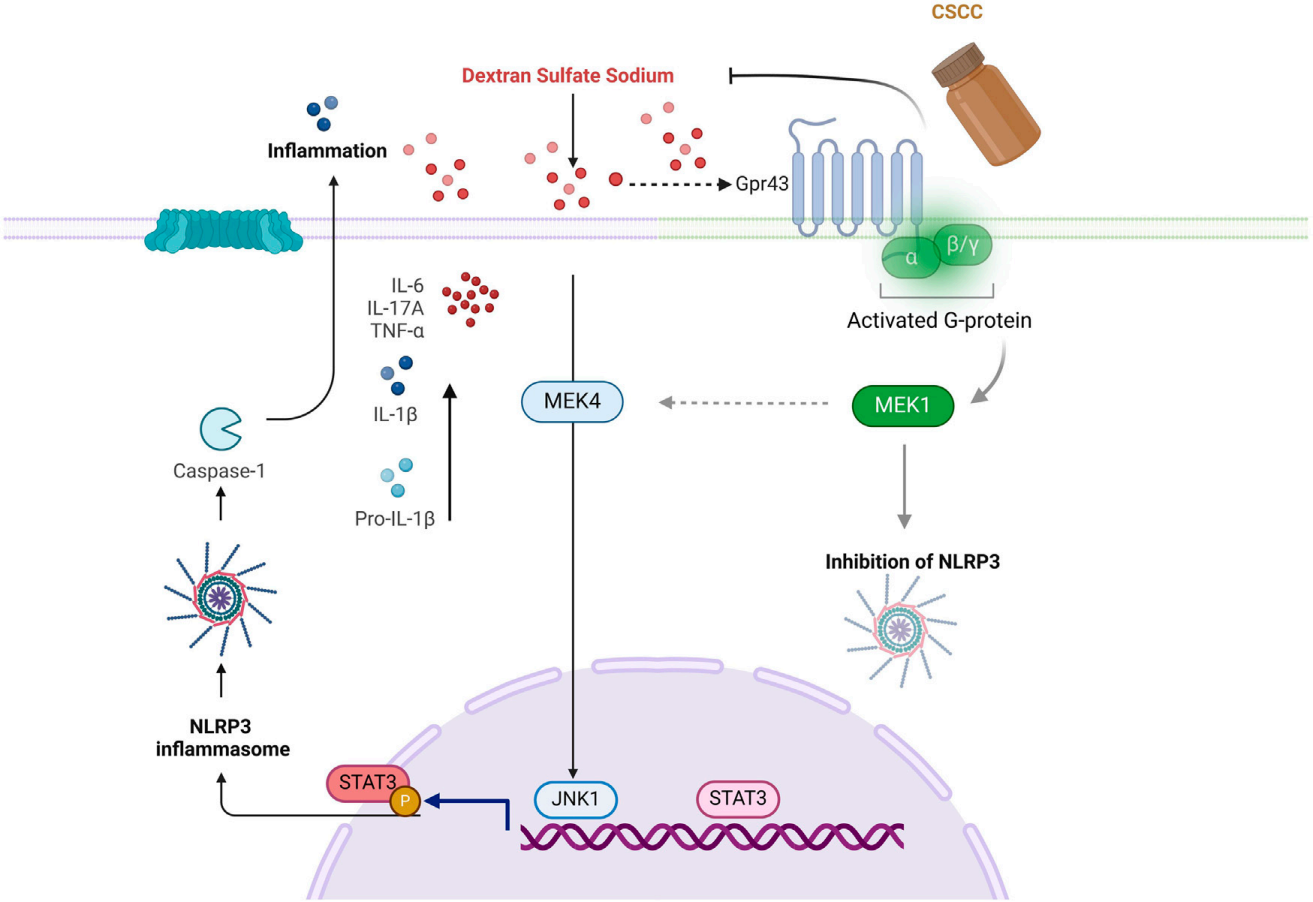
Composite Sophora Colon-soluble Capsule upregulated Gpr43 to inhibit the STAT3/NLRP3 pathway and suppress inflammation factors.

In conclusion, our findings suggest that CSCC treatment can protect mice from DSS-induced colitis by upregulating Gpr43, promoting the expression of ZO-1 and Occludin tight junction proteins. Mechanistically, CSCC inhibits the MEK4/JNK1/STAT3 activation pathway, consequently suppressing the STAT3/NLRP3/IL-1β pathway and inhibiting the production of inflammatory factors such as IL-17A (Figure 7). This highlights the mechanisms by which CSCC exerts its anti-colitis effects. This study indicates that CSCC may serve as a potential therapeutic candidate for controlling the progression of UC.

## Data Availability

The original contributions presented in the study are included in the article/Supplementary Material, further inquiries can be directed to the corresponding author.

## Abbreviations

5-ASA, mesalazine; BSA, bovine serum albumin; CSCC, Wuwei Kushen Changrong capsule; DMEM, Dulbecco’s Modified Eagle Medium; DSS, Dextran sulfate sodium ECCO, European Crohn’s and Colitis Organization; ECL, electrochemiluminescence; ERK, extracellular signal-regulated kinase; FBS, fetal bovine serum; GM-CSF, granulocyte-macrophage colony-stimulating factor; GPCR, G protein-coupled receptor; Gpr43; G protein-coupled receptor 43; IL, interleukin; LPS, lipopolysaccharide; MEK1, mitogen-activated protein kinase 1; NLRP3, NACHT, LRR, and PYD domains-containing protein 3; PAGE, polyacrylamide gel electrophoresis; PBS, phosphate-buffered saline; PFA, paraformaldehyde; P-STAT3, phosphorylated transducer and activator of transcription-3; PVDF, polyvinylidene fluoride; RIPA, radioimmunoprecipitation assay; SDS, sodium dodecyl sulfate; SFCA, short-chain fatty acids; TBST, tris-buffered saline and Polysorbate 20; TCM, traditional Chinese medicine; TEMED, tetramethylethylenediamine; TNF-α, tumor necrosis factor alpha; UC, ulcerative colitis; UHPLC-Q-TOF/MS, ultra-high performance liquid chromatography-quadruple time-of-flight mass spectrometry; XBP1, X-box binding protein 1.
